# Estimated glucose disposal rate as a candidate biomarker for thrombotic biomarkers in T1D: a pooled analysis

**DOI:** 10.1007/s40618-021-01550-3

**Published:** 2021-03-17

**Authors:** L. L. O’Mahoney, N. Kietsiriroje, S. Pearson, D. J. West, M. Holmes, R. A. Ajjan, M. D. Campbell

**Affiliations:** 1 Diabetes Research Centre, Leicester General Hospital, University of Leicester, Leicester, UK; 2 Carnegie School of Sport, Leeds Beckett University, Leeds, UK; 3 Endocrinology and Metabolism Unit, Faculty of Medicine, Prince of Songkla University, Songkhla, Thailand; 4 University of Leeds, Leeds Institute for Cardiovascular and Metabolic Medicine, Leeds, UK; 5 Human Nutrition Research Centre, Newcastle University, Newcastle, UK; 6 Faculty of Medical Science, Newcastle University, Population Health Science Institute, Newcastle, UK; 7 School of Food Science and Nutrition, University of Leeds, Leeds, UK; 8 School of Food Science and Biotechnology, Zhejiang Gongshang University, Hangzhou, China; 9 University of Sunderland, Institute of Health Sciences and Wellbeing, Sunderland, UK

**Keywords:** eGDR, Type 1 diabetes, Thrombosis, Cluster analysis

## Abstract

**Purpose:**

To determine the utility of estimated glucose disposal rate (eGDR) as a candidate biomarker for thrombotic biomarkers in patients with type 1 diabetes (T1D).

**Methods:**

We reanalysed baseline pretreatment data in a subset of patients with T1D from two previous RCTs, consisting of a panel of thrombotic markers, including fibrinogen, tissue factor (TF) activity, and plasminogen-activator inhibitor (PAI)-1, and TNFα, and clinical factors (age, T1D duration, HbA1c, insulin requirements, BMI, blood pressure, and eGDR). We employed univariate linear regression models to investigate associations between clinical parameters and eGDR with thrombotic biomarkers.

**Results:**

Thirty-two patients were included [mean ± SD age 31 ± 7 years, HbA1c of 58 ± 9 mmol/mol (7.5 ± 0.8%), eGDR 7.73 ± 2.61]. eGDR negatively associated with fibrinogen (*P* < 0.001), PAI-1 concentrations (*P* = 0.005), and TF activity (*P* = 0.020), but not TNFα levels (*P* = 0.881). We identified 2 clusters of patients displaying significantly different characteristics; 56% (*n* = 18) were categorised as ‘higher-risk’, eliciting significantly higher fibrinogen (+ 1514 ± 594 μg/mL; *P* < 0.001), TF activity (+ 59.23 ± 9.42 pmol/mL; *P* < 0.001), and PAI-1 (+ 8.48 ± 1.58 pmol/dL; *P* < 0.001), HbA1c concentrations (+ 14.20 ± 1.04 mmol/mol; *P* < 0.001), age (+ 7 ± 3 years; *P* < 0.001), duration of diabetes (15 ± 2 years; *P* < 0.001), BMI (+ 7.66 ± 2.61 kg/m^2^; *P* < 0.001), and lower mean eGDR (− 3.98 ± 1.07; *P* < 0.001).

**Conclusions:**

Compared to BMI and insulin requirements, classical surrogates of insulin resistance, eGDR is a suitable and superior thrombotic risk indicator in T1D.

**Trial registration:**

ISRCTN4081115; registered 27 June 2017.

**Supplementary Information:**

The online version contains supplementary material available at 10.1007/s40618-021-01550-3.

## Introduction

Insulin resistance in type 1 diabetes (T1D) is an established risk factor for cardiovascular disease [1, 2], retinopathy [3], and premature mortality [4]. The pathological linkage between insulin resistance and increased vascular risk is largely in virtue of an enhanced prothrombotic milieu [5–8]. Under normal conditions, insulin inhibits platelet aggregation and thrombosis via tissue factor (TF) inhibition and enhanced fibrinolytic action due to modulation of plasminogen activator inhibitor-1 (PAI-1) [6]. In contrast, both T1D [9] and insulin resistance are associated with a procoagulant plasma profile [10], whereby increased PA1-1 and fibrinogen and reduced tissue plasminogen activator promotes atherothrombosis [11]. As such, identifying and treating insulin resistance in people with T1D represents an important therapeutic goal in reducing thrombotic biomarkers and preventing the development of overt vascular complications.

However, the gold-standard technique for identifying and quantifying insulin resistance—the euglycaemic hyperinsulinaemic clamp—is time-consuming and invasive, rendering it impractical for use in routine clinical settings. Further, the use of individual clinical parameters in isolation, such as body mass index (BMI) or insulin dose requirements are crude indicators of insulin resistance in T1D. An alternative, is the use of estimated glucose disposal rate (eGDR), a validated marker of insulin resistance [12–14] which uses a combination of clinical parameters including HbA1c, BMI, and the presence of hypertension [15]. Research investigating the utility of eGDR has shown this metric to be associated with nephropathy [16] peripheral vascular disease [17], coronary artery disease [18, 19], and mortality [19] with lower eGDR values conferring greater risk. In the present study, we reanalysed data in a subset of patients with T1D from two RCTs and sought to explore the utility of eGDR as a candidate biomarker specifically for thrombotic biomarkers. Further, we applied an unsupervised, data-driven cluster analysis, to establish a novel classification for thrombosis in our cohort, based on shared commonalities between routine clinical parameters and thrombotic biomarkers.

## Methods

We used data from two previous RCTs (Clinical trial registration: clinicaltrials.gov NCT02595658; ISRCTN registration ISRCTN40811115). Both studies received ethical approval from local National Health Service Research Ethics Committees (REC reference 14/NE/1183; REC reference 17/NE/0244) and all participants gave written informed consent.

Detailed information regarding each study has been published previously [9, 20]. In the present analysis, we included participants meeting the following inclusion criteria: classical presentation of T1D (including primary osmotic symptoms, weight loss, hyperglycaemia, ketosis, insulin initiation at diagnosis); aged 18–50 years; diagnosed with T1D for a minimum of 5-years on enrolment; treated on a stable (> 12-months) basal-bolus insulin regimen consisting of rapid-acting insulin analogues lispro or aspart and basal insulin glargine delivered through multiple daily injections or continuous subcutaneous insulin infusion; and free of diabetes-related complications.

We used baseline pretreatment data across both RCTs. In both studies, testing procedures were conducted during a morning-time laboratory visit with patients adopting an overnight fast (> 10-h). Fasted, rested, venous blood samples were obtained and retrospectively analysed for, tumour necrosis factor alpha (TNFα; Human TNFα Quantikine ELISA; R and D Systems, Roche Diagnostics, UK), plasma fibrinogen (ab108842, Fibrinogen Human ELISA Kit; Abcam, Japan), tissue factor activity (TF; Human Tissue Factor activity ab108906; Abcam, UK) and plasminogen activator inhibitor-1 (PAI-1; Human PAI-1/serpin ELISA Kit DSE100; R and D systems, UK) using methods previously described [9]; the intra-assay coefficient of variation was < 10% for all biochemical analysis. In addition, we obtained the following physiological characteristics (age, duration of diabetes, HbA1c, insulin requirements, BMI, blood pressure, and eGDR). Blood pressure was assessed via an automated oscillometric device (Intellisense HEM-907XL, Omron, Japan); participants were categorised as hypertensive if ≥ 140/90mmHG, pre-existing physicians’ diagnosis, or antihypertensive use [21]. eGDR was calculated using a composite of BMI, HbA1c and hypertensive status using the following formulae: eGDR = 19.02—[0.22 X BMI (kg/m^2^)]—(3.26 X HTN)—(0.61 X HbA1c (%)], whereby HTN is hypertension (1 = yes, 0 = no). eGDR was used as a diagnostic criterion for insulin resistance with lower eGDR values conferring greater degrees of insulin resistance [15].

### Statistical analysis

Data were analysed using SPSS Statistics version 25 (IBM SPSS Statistics 25, IBM Corporation, USA) and checked for normality using Shaprio–Wilk’s test with a cut-point 0.05. Descriptive characteristics of the study population are presented as mean ± SD for continuous variables and as frequency (%) for categorical variables; 95% confidence intervals (CIs) and *β* coefficients are presented where relevant. To assess the association between clinical parameters and eGDR, a Pearson correlation coefficient matrix was employed. Univariate linear regression models were used to investigate the associations between clinical parameters and thrombotic biomarkers. To categorise and group individuals based on shared clinical and biochemical characteristics, we utilised two-step clustering with complete data available for continuous variables. In this unsupervised approach, the first step estimates the optimal number of clusters on the basis of silhouette width and the second step is based on Bayesian hierarchical clustering. In this application, the method partitions clinical characteristics based on their abundance/magnitude in the individuals, and partitions individuals based on the abundance/magnitude of their characteristics. The optimal number of clusters was determined to be 2. We use standardised Z scores of variables and log-likelihood as a distance measure and Schwarz’s Bayesian criterion for clustering. Only continuous variables were included as the k-means method does not accommodate binary categorical variables. Cluster labels were assigned by examining cluster variable means. Differences between dichotomised variables were assessed with independent *t*-tests. Statistical significance was set at *P* < 0.05 for all analyses.

## Results

**Fig. 1:**
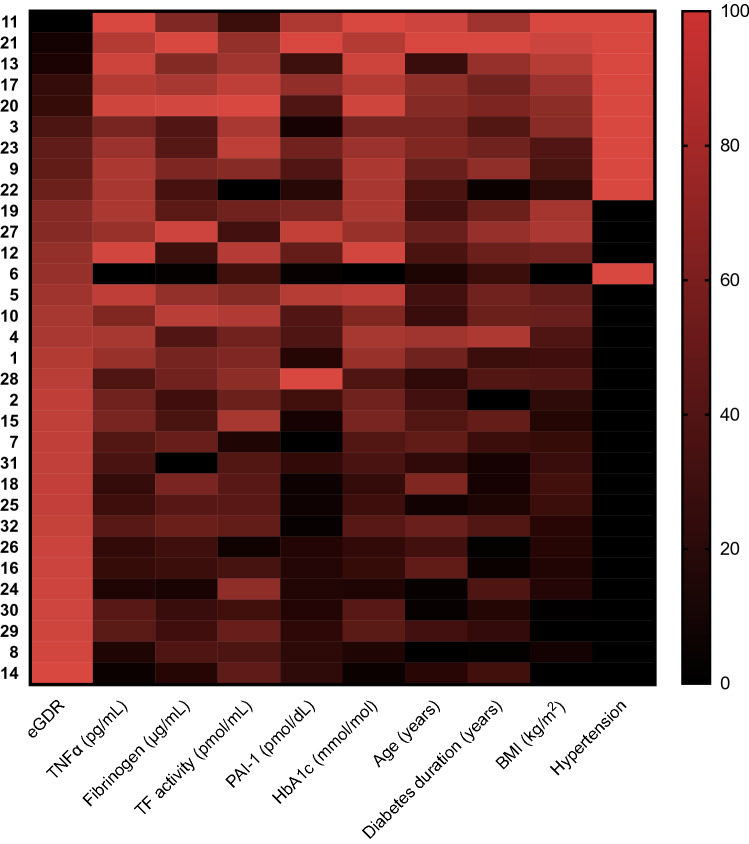
Individual patient clinical profiles (y axis) ranked by eGDR (normalised data). eGDR, estimated glucose disposal rate; TF activity, Tissue Factor activity; PAI-1, Plasminogen Activator Inhibitor-1; TNFα, Tumour Necrosis Factor alpha

**Fig. 2:**
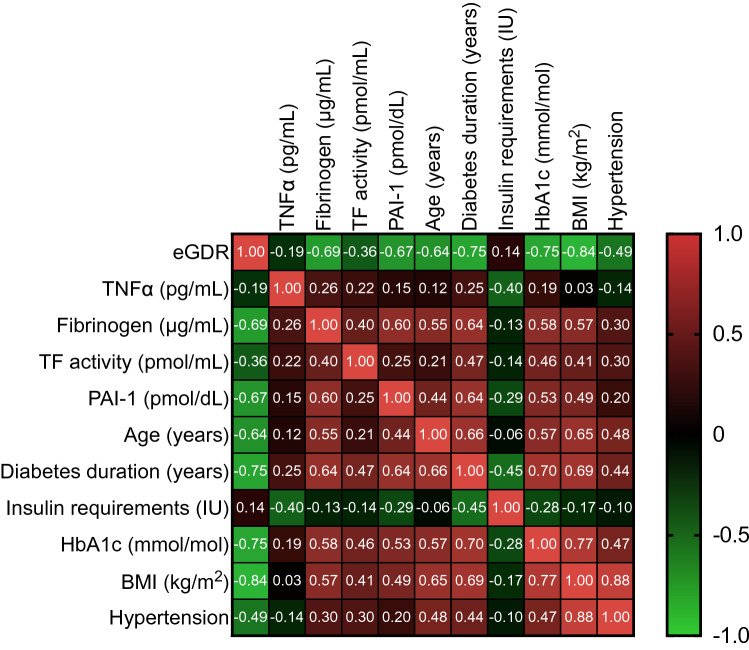
Pearson correlation coefficient matrix illustrating the association between baseline patient characteristics and eGDR. Pearson correlation coefficients (*r*) are highlighted in white text. eGDR, estimated Glucose Disposal Rate; TF activity, Tissue Factor activity; PAI-1, Plasminogen Activator Inhibitor-1; TNFα, Tumour Necrosis Factor alpha

Baseline characteristics of patients included in the present analysis are shown in Table 1. In summary, the 32 T1D males had a mean age of 31 ± 7 years, HbA1c of 58 ± 9 mmol/mol [7.5 ± 0.8%], and a mean eGDR value of 7.73 ± 2.61. Figure 1 shows individual patient clinical profiles ranked by eGDR. To determine whether, and identify which, clinical parameters may serve as candidate biomarkers for a thrombotic biomarker profile, we applied a Pearson correlation coefficient matrix across variables (Fig. 2). eGDR was negatively correlated with fibrinogen (*r* = − 0.69; *P* < 0.001), PAI-1 concentrations (*r* = − 0.67; *P* = 0.005), and TF activity (*r* = − 0.36; *P* = 0.020), but not TNFα levels (*r* = − 0.19; *P* = 0.881). HbA1c, BMI, age, diabetes duration, and insulin requirements were positively correlated with thrombotic biomarkers (Fig. 2). The relationship between thrombotic biomarkers and clinical characteristics was further examined using univariate regression analysis (Table 2). eGDR, HbA1c, BMI, age, diabetes duration, and insulin requirements were significantly associated with fibrinogen, TF activity, and PAI-1 concentrations, with eGDR providing the strongest association across the range of thrombotic biomarkers (Table 2). Multivariate modelling to assess for the contribution of the relationship between significant associations could not be performed due to the small numbers of this study.

**Table 1:** Baseline characteristics of patients

Clinical parameters	
Age (years)	31 ± 7 [21–50]
BMI (kg/m^2^)	26.03 ± 4.82 [20–38]
Egdr	7.73 ± 2.61 [2.12–10.72]
Hypertension (%)	31
HbA1c [mmol.moL (%)]	57.97 ± 8.85 (7.45 ± 0.81) 43.17–72.64 (6.10–8.80)
Diabetes duration (years)	17 ± 9 [4–42]
Total daily insulin requirements (IU)	42 ± 2 [83–8]
Rapid-acting insulin requirements (IU)	9 ± 3 [4–16]
Insulin apart users (%)	63
Vascular and inflammatory parameters	
TNFα (pg/mL)	4.28 ± 1.05 [2.30–6.01]
Fibrinogen (μg/mL)	2221 ± 1238 [300–5060]
TF activity (pmol/mL)	108.93 ± 52.20 [11.26–219.52]
PAI-1 (pmol/dL)	12.33 ± 7.50 [4.48–30.09]

Metric variables presented as mean ± SD [Range]; categorical data presented as frequency (%)

*eGDR* estimated glucose disposal rate, *TF activity* tissue factor activity, *PAI-1* Plasminogen activator inhibitor-1, *TNFα* tumour necrosis factor alpha

**Table 2:** Linear regression analysis of thrombotic biomarkers with clinical parameters

	Fibrinogen			TF activity			PAI-1		
	*β* (95% CI)	*R*^2^	*P* value	*β* (95% CI)	*R*^2^	*P* value	*β* (95% CI)	*R*^2^	*P* value
Age (years)	0.003 (0.001, 0.005)	0.31	0.001**	0.028 (− 0.021, 0.076)	0.04	0.025*	4.077 (1.011, 7.144)	0.20	0.011*
BMI (kg/m^2^)	0.001 (0.001, 0.002)	0.33	< 0.001***	0.021 (− 0.000, 0.038)	0.17	0.020*	1.695 (0.560, 2.830)	0.24	0.005**
eGDR	− 0.003 (− 0.004, − 0.002)	0.48	< 0.001***	0.084 (0.025, 0.142)	0.21	0.007**	− 4.284 (− 6.071, − 2.496)	0.44	< 0.001***
Hypertension (%)	0.000 (− 1.980, 0.000)	0.09	0.092	0.003 (0.000, 0.006)	0.09	0.100	0.125 (− 0.105, 0.354)	0.04	0.276
HbA1c (mmol.moL [%])	0.004 (0.002,0.016)	0.34	< 0.001***	0.078 (0.022, 0.134)	0.21	0.008**	6.211 (2.473, 9.948)	0.28	0.002**
Diabetes duration (years)	0.005 (0.003, 0.007)	0.41	< 0.001***	− 0.034 (0.066, − 0.002)	0.13	0.040*	7.961 (4.419, 11.500)	0.41	< 0.001***
Insulin requirements (IU)	0.001 (0.000, 0.002)	0.23	< 0.006**	0.023 (0.004, 0.042)	0.17	0.018*	1.523 (0.207, 2.840)	0.16	0.025*

*eGDR* estimated glucose disposal rate, *TF activity* tissue factor activity, *PAI-1* plasminogen activator inhibitor-1, *TNFα* tumour necrosis factor alpha

*P < 0.05

**P < 0.01

***P < 0.001

**Fig. 3:**
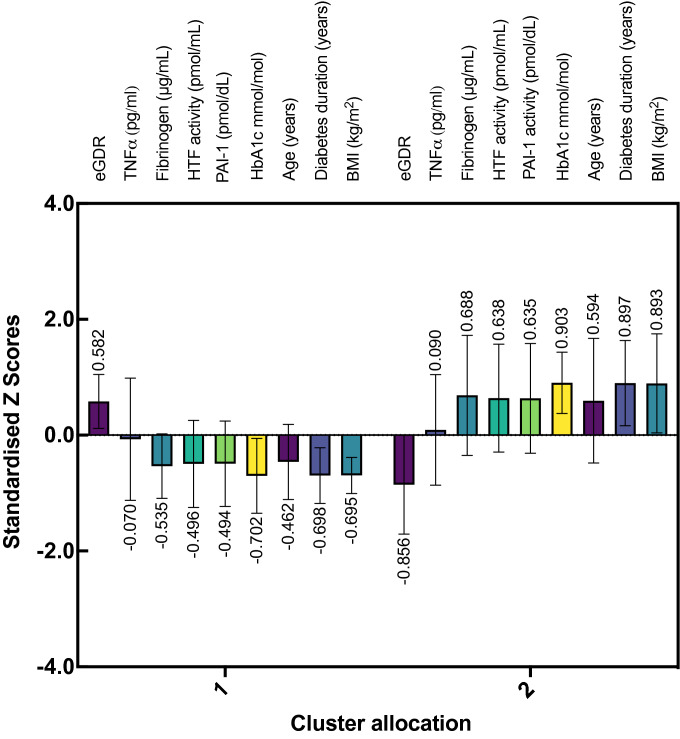
Cluster characteristics. Variables are presented as standardised Z scores. eGDR, estimated Glucose Disposal Rate; TF activity, Tissue Factor activity; PAI-1, Plasminogen Activator Inhibitor-1; TNFα, Tumour Necrosis Factor alpha

**Fig. 4:**
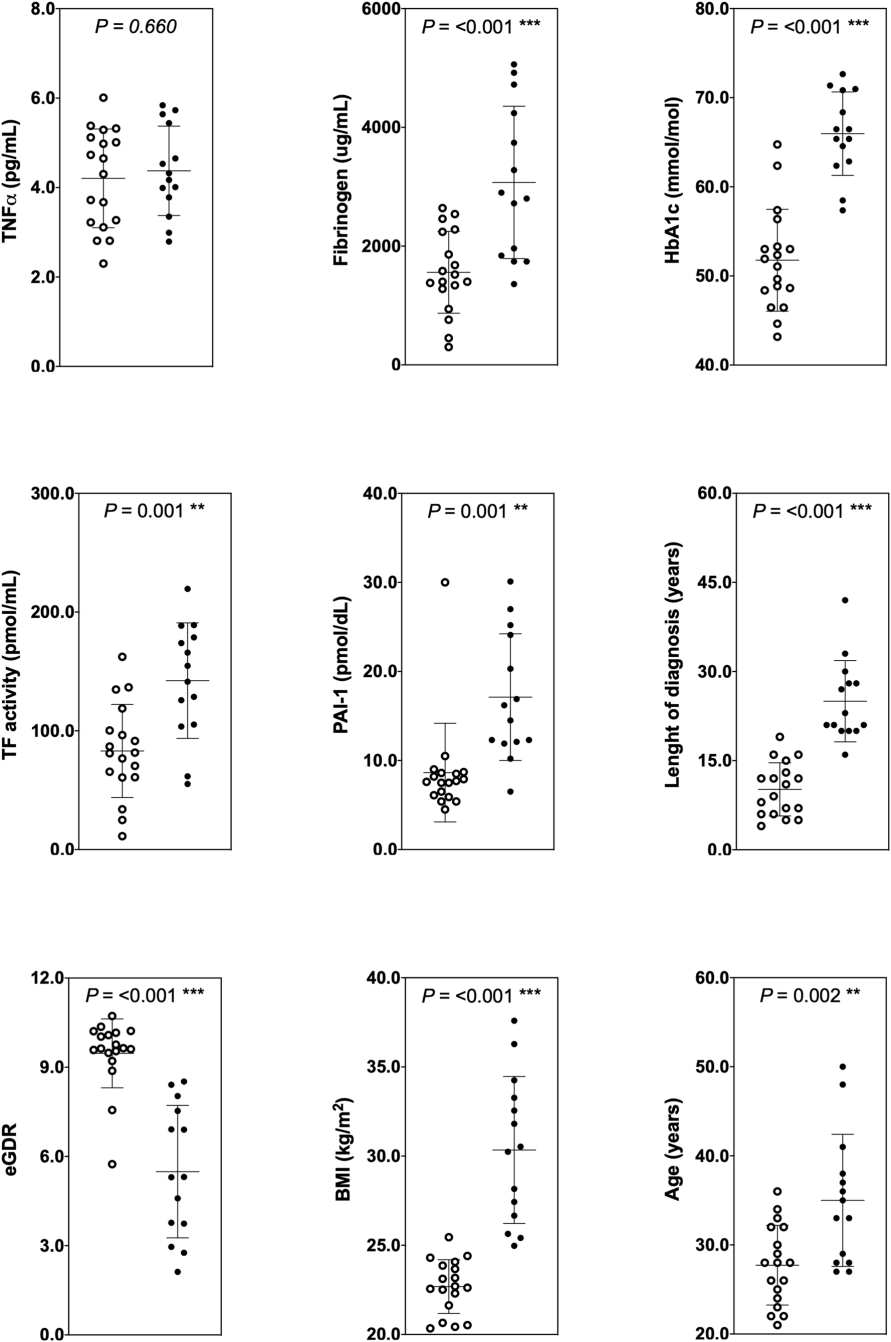
Patient characteristics stratified by cluster allocation. White circles = cluster 1; black circles = cluster 2. Statistically significant differences between clusters calculated using independent *t*-tests. * denotes *P* < 0.05; ** denotes *P* < 0.01; *** denotes *P* < 0.001. *eGDR* estimated glucose disposal rate, *TF activity* tissue factor activity, *PAI-1* plasminogen activator inhibitor-1, *TNFα* tumour necrosis factor alpha

To identify whether clinical profiles could be used to classify patients into novel diabetes subgroups, we used a two-step clustering method with the complete data available for continuous clustering variables. Figure 3 shows the cluster characteristics for cluster 1 and 2. Cluster 2, including 44% (*n* = 14) of the patients, was characterised by increased levels of vascular inflammatory proteins: fibrinogen, TF activity, PAI-1, and their mediator TNFα, a higher HbA1c, older age, a greater duration of diabetes, and increased BMI, and lower eGDR. These data imply that patients with a lower eGDR, a proxy of insulin resistance, concomitantly express raised levels of thrombotic biomarkers associated with adverse vascular health. To explore this hypothesis, we stratified patients according to their cluster allocation and performed independent *t*-tests on individual clinical parameters and thrombotic biomarkers (Fig. 4). Clinical parameters and thrombotic biomarkers of patients stratified by cluster allocation can found in Online Resource 1. Notably, cluster 2, was categorised as a ‘higher-thrombotic profile’ group, with individuals eliciting significantly higher mean fibrinogen (cluster 1: 1559 ± 689 vs. cluster 2: 3073 ± 1283 μg/mL; *P* < 0.001), TF activity (cluster 1: 83.01 ± 39.20 vs. cluster 2: 142.24 ± 48.62 pmol/mL; *P* = 0.001), and PAI-1 (cluster 1: 8.62 ± 5.53 vs. cluster 2: 17.10 ± 7.11 pmol/dL; *P* = 0.001), HbA1c concentrations (cluster 1: 51.76 ± 5.72 vs. cluster 2: 65.96 ± 4.68 mmol/mol; *P* < 0.001). Further, cluster 2, elicited a significantly higher mean age (cluster 1: 28 ± 5 vs. cluster 2: 35 ± 7 years; *P* = 0.002), greater mean duration of diabetes (cluster 1: 10 ± 5 vs. cluster 2: 25 ± 7 years; *P* < 0.001), a higher mean BMI (cluster 1: 22.68 ± 1.51 vs. cluster 2: 30.34 ± 4.12 kg/m^2^; *P* < 0.001), and a lower mean eGDR (cluster 1: 9.47 ± 1.16 vs. cluster 2: 5.49 ± 2.23; *P* < 0.001).

## Conclusions

In the present study, we examined, for the first time, the association between eGDR, a validated surrogate marker of insulin resistance, and thrombotic biomarkers in patients with T1D. Our findings suggest eGDR to be a suitable indicator of a prothrombotic profile, and superior to BMI and insulin requirements which are classical surrogates of insulin resistance.

Whereas, previous attempts to assess the relationship between eGDR and vascular health have focused on established microvascular complications [22], to the best of our knowledge, this is the first study that has aimed to assess whether, and which, clinical characteristics may serve as candidate biomarkers specifically for a heightened thrombotic profile in the absence of established microvascular complications. Consequently, our data lend support to the use of eGDR as a tool for identifying T1D patients with a procoagulant profile prior to the presentation of overt vascular complications. Further, established risk factors for complications in T1D, namely HbA1c, age, and disease duration [23–25] were also found to be associated with an adverse thrombotic profile in this group of relatively young, and well-controlled T1D adults. In addition to these classical risk factors, we propose that eGDR may serve as a useful clinical tool for targeting individuals requiring closer monitoring for early atherothrombosis and potential intervention for insulin resistance.

As our data imply that patients with a lower eGDR, older age, longer duration of diabetes, and higher insulin requirements, concomitantly express a procoagulant profile, a logical extension of the present study was to establish whether it was possible to classify patients based on shared commonalities in clinical characteristics and thrombotic biomarkers. To this end, we applied an unsupervised, data-driven cluster analysis to establish a novel classification for an elevated thrombotic profile in T1D. Importantly, we used variables reflective of key aspects of diabetes management that can be easily obtained and monitored in patients, and thus this clustering can implemented in both existing diabetes cohorts and patients in diabetes clinics. Within the ‘higher-thrombotic profile’ cluster, circulating concentrations of fibrinogen, TF activity, and PAI-1 were on average ~ twofold higher than levels exhibited by patients in the ‘lower-thrombotic profile’ cluster. This was accompanied by ~ twofold lower eGDR score in the ‘higher-thrombotic profile’ cluster [eGDR: cluster 1 (lower-thrombotic profile) ~ 5.5 vs. cluster 2 (higher-thrombotic profile) ~ 9.5] which is characteristic of a ‘double diabetes’ phenotype [15]—a cohort at increased cardiovascular risk [14]. For example, in a large longitudinal cohort study, Nyström and colleagues [14] recently demonstrated an eGDR of eight or less to be associated with an increased risk of cardiovascular disease or death in individuals with T1D compared to individuals with an eGDR greater than eight where survival rates were identical to a matched reference population.

It is well established that an enhanced thrombotic environment contributes to poor clinical outcomes in patients with diabetes [26]. TF activity levels are increased in people with diabetes, which upregulates production of thrombin accelerating the risk of clot formation [6]. Moreover, raised fibrinogen concentrations, reflective of chronic low-grade inflammation contributes to the formation of denser clots, and increased PAI-1 levels impair the fibrinolytic process [6]. Previous in vitro and in vivo studies have shown that individuals with type 2 diabetes, but not necessarily T1D exhibit increased PAI-1 levels suggesting that insulin resistance rather than just hyperglycaemia per se, promotes increased antifibrinolytic protein production [27]. This would suggest that targeting insulin resistance specifically, and not just glycaemia, may have an important effect on PAI-1 levels. PAI-1 levels in our patients were, on average comparable to previous reports in complication-free T1D individuals [28]. However, we observed a large range in PAI-1 levels between our patients, with some individuals exhibiting levels similar to patients with type 2 diabetes [29]. Taken collectively, our findings highlight a large degree of heterogeneity in the presentation of thrombotic biomarkers between T1D patients, which highlights the complexities involved in the management of elevated thrombotic profiles in this patient group and questions the appropriateness of managing T1D uniformly.

From this initial exploratory study, we cannot at this stage claim that the clusters present different and distinct phenotypes of T1D, or that the clustering applied herein is the optimal classification of an elevated thrombotic profile across the spectrum of T1D. Future prospective studies with larger cohorts should focus attempts on addressing this aim to refine stratification through the inclusion of additional cluster variables, such as genotypes, or genetic risk scores, and, to establish whether patients can move between clusters in response to therapy or intervention, and apply resampling methods to derive the significance of identified groups. Limitations of this work include not screening for C-peptide or autoantibodies and therefore we cannot rule out the possibility of a T1D misdiagnosis [i.e. maturity on the basis of classical presentation (including primary osmotic symptoms, weight loss, hyperglycaemia, ketosis, insulin initiation at diagnosis)], and our relatively limited sample consisting of relatively young well-controlled patients.

In conclusion, our findings suggest eGDR to be a suitable tool for routine clinical practice for identifying T1D patients with a procoagulant profile prior to the presentation of overt vascular complications.

## Supplementary Information

Below is the link to the electronic supplementary material.

Supplementary file1 (DOCX 18 KB)

## Data Availability

The data that support the findings of this study are available on request from the corresponding author.

## References

[1] Donga E, Dekkers OM, Corssmit E, Romijn JA (2015). Insulin resistance in patients with type 1 diabetes assessed by glucose clamp studies: systematic review and meta-analysis. Eur J Endocrinol.

[2] Schauer IE, Snell-Bergeon JK, Bergman BC, Maahs DM, Kretowski A, Eckel RH, Rewers M (2011). Insulin resistance, defective insulin-mediated fatty acid suppression, and coronary artery calcification in subjects with and without type 1 diabetes: the CACTI study. Diabetes.

[3] Chaturvedi N, Sjoelie A-K, Porta M, Aldington SJ, Fuller JH, Songini M, Kohner EM (2001). Markers of insulin resistance are strong risk factors for retinopathy incidence in type 1 diabetes: the EURODIAB prospective complications study. Diabetes Care.

[4] Mäkinen V-P, Forsblom C, Thorn LM, Wadén J, Gordin D, Heikkilä O, Hietala K, Kyllönen L, Kytö J, Rosengård-Bärlund M (2008). Metabolic phenotypes, vascular complications, and premature deaths in a population of 4,197 patients with type 1 diabetes. Diabetes.

[5] Members ATF, Rydén L, Grant PJ, Anker SD, Berne C, Cosentino F, Danchin N, Deaton C, Escaned J, Hammes H-P (2013). ESC guidelines on diabetes, pre-diabetes, and cardiovascular diseases developed in collaboration with the EASD: the task force on diabetes, pre-diabetes, and cardiovascular diseases of the European Society of Cardiology (ESC) and developed in collaboration with the European Association for the Study of Diabetes (EASD). Eur Heart J.

[6] Kearney K, Tomlinson D, Smith K, Ajjan R (2017). Hypofibrinolysis in diabetes: a therapeutic target for the reduction of cardiovascular risk. Cardiovasc Diabetol.

[7] Grant P (2007). Diabetes mellitus as a prothrombotic condition. J Intern Med.

[8] Vazzana N, Ranalli P, Cuccurullo C (2012). DavıG mellitus and thrombosis. Thromb Res.

[9] Campbell MD, Walker M, Ajjan RA, Birch KM, Gonzalez JT, West DJ (2017). An additional bolus of rapid-acting insulin to normalise postprandial cardiovascular risk factors following a high-carbohydrate high-fat meal in patients with type 1 diabetes: a randomised controlled trial. Diab Vasc Dis Res.

[10] Ozkul A, Turgut ET, Akyol A, Yenisey C, Kadikoylu G, Tataroglu C, Kiylioglu N (2010). The relationship between insulin resistance and hypercoagulability in acute ischemic stroke. Eur Neurol.

[11] Paneni F, Beckman JA, Creager MA, Cosentino F (2013). Diabetes and vascular disease: pathophysiology, clinical consequences, and medical therapy: part I. Eur Heart J.

[12] Williams KV, Erbey JR, Becker D, Arslanian S, Orchard TJ (2000). Can clinical factors estimate insulin resistance in type 1 diabetes?. Diabetes.

[13] Epstein EJ, Osman JL, Cohen HW, Rajpathak SN, Lewis O, Crandall JP (2013). Use of the estimated glucose disposal rate as a measure of insulin resistance in an urban multiethnic population with type 1 diabetes. Diabetes Care.

[14] Nyström T, Holzmann MJ, Eliasson B, Svensson AM, Sartipy U (2018). Estimated glucose disposal rate predicts mortality in adults with type 1 diabetes. Diabetes Obes Metab.

[15] Kietsiriroje N, Pearson S, Campbell M, Ariëns RA, Ajjan RA (2019). Double diabetes: a distinct high-risk group?. Diabetes Obes Metab.

[16] Orchard TJ, Chang Y-F, Ferrell RE, Petro N, Ellis DE (2002). Nephropathy in type 1 diabetes: a manifestation of insulin resistance and multiple genetic susceptibilities? Further evidence from the Pittsburgh Epidemiology of Diabetes Complication Study. Kidney Int.

[17] Olson JC, Erbey JR, Forrest KY, Williams K, Becker DJ, Orchard TJ (2002). Glycemia (or, in women, estimated glucose disposal rate) predict lower extremity arterial disease events in type 1 diabetes. Metabol Clin Exp.

[18] Orchard TJ, Olson JC, Erbey JR, Williams K, Forrest KY-Z, Kinder LS, Ellis D, Becker DJ (2003). Insulin resistance–related factors, but not glycemia, predict coronary artery disease in type 1 diabetes: 10-year follow-up data from the Pittsburgh Epidemiology of Diabetes Complications study. Diabetes Care.

[19] Pambianco G, Costacou T, Orchard TJ (2007). The prediction of major outcomes of type 1 diabetes: a 12-year prospective evaluation of three separate definitions of the metabolic syndrome and their components and estimated glucose disposal rate: the Pittsburgh Epidemiology of Diabetes Complications Study experience. Diabetes Care.

[20] O'Mahoney LL, Dunseath G, Churm R, Holmes M, Boesch C, Stavropoulos-Kalinoglou A, Ajjan RA, Birch KM, Orsi NM, Mappa G, Price OJ, Campbell MD (2020). Omega-3 polyunsaturated fatty acid supplementation versus placebo on vascular health, glycaemic control, and metabolic parameters in people with type 1 diabetes: a randomised controlled preliminary trial. Cardiovasc Diabetol.

[21] NICE (2019) Hypertension in adults: diagnosis and management

[22] NICE guideline [NG136]. https://www.nice.org.uk/guidance/ng136. Accessed 12/05/2020

[23] Girgis CM, Scalley BD, Park KE (2012). Utility of the estimated glucose disposal rate as a marker of microvascular complications in young adults with type 1 diabetes. Diabetes Res Clin Pract.

[24] Nazaimoon WW, Letchuman R, Noraini N, Ropilah A, Zainal M, Ismail I, Mohamad WW, Faridah I, Singaraveloo M, Sheriff I (1999). Systolic hypertension and duration of diabetes mellitus are important determinants of retinopathy and microalbuminuria in young diabetics. Diabetes Res Clin Pract.

[25] Rodrigues T, Canani L, Schvartzman P, Gross J (2011). Hypertension is the metabolic syndrome component most strongly associated with microvascular complications and coronary artery calcification in type 1 diabetes. J Endocrinol Invest.

[26] Nørgaard K, Feldt-Rasmussen B, Deckert T (1991). Is hypertension a major independent risk factor for retinopathy in type 1 diabetes?. Diabet Med.

[27] Sumaya W, Wallentin L, James SK, Siegbahn A, Gabrysch K, Bertilsson M, Himmelmann A, Ajjan RA (2018). Fibrin clot properties independently predict adverse clinical outcome following acute coronary syndrome: a PLATO substudy. Eur Heart J.

[28] King R, Ajjan R (2016) Hypoglycaemia, thrombosis and vascular events in diabetes. Taylor and Francis 10.1080/14779072.2016.1215916 27460231

[29] Adly AAM, Elbarbary NS, Ismail EAR, Hassan SR (2014). Plasminogen activator inhibitor-1 (PAI-1) in children and adolescents with type 1 diabetes mellitus: relation to diabetic micro-vascular complications and carotid intima media thickness. JDC.

[30] Yarmolinsky J, Barbieri NB, Weinmann T, Ziegelmann PK, Duncan BB, Schmidt MI (2016). Plasminogen activator inhibitor-1 and type 2 diabetes: a systematic review and meta-analysis of observational studies. Sci Rep.

