# Supplementary material for: Estimated glucose disposal rate as a candidate biomarker for thrombotic biomarkers in T1D: a pooled analysis
Source: J Endocrinol Invest. 2021 Mar 17;44(11):2417–26. doi: 10.1007/s40618-021-01550-3 (PMC8502148; doi:10.1007/s40618-021-01550-3)
Supplement: Supplementary file 1 — Supplementary file1 (DOCX 18 KB) [file 40618_2021_1550_MOESM1_ESM.docx]

**SUPPLEMENTARY INFORMATION**

| **Table 1.** Clinical parameters and thrombotic biomarkers in patients stratified by cluster allocation | | | |
| --- | --- | --- | --- |
|  | **Cluster 1** | **Cluster 2** | ***P*-value** |
| TNFα (pg/mL) | 4.21±1.10 | 4.37±1.00 | =0.660 |
| Fibrinogen (ug/mL) | 1559±689 | 3073±1283 | <0.001 |
| TF activity (pmol/mL) | 83.01±39.20 | 142.24±48.62 | =0.001 |
| PAI-1 (pmol/dL) | 8.62±5.53 | 17.10±7.11 | =0.001 |
| HbA1c (mmol/mol) | 51.76±5.72 | 65.96±4.86 | <0.001 |
| Length of diabetes (years) | 10±4 | 25±7 | <0.001 |
| eGDR | 9.47±1.16 | 5.49±2.23 | <0.001 |
| BMI (kg/m^2^) | 22.68±1.58 | 30.34±4.12 | <0.001 |
| Age (years) | 28±4 | 35±7 | =0.002 |
| Data presented as mean±SD. Differences between dichotomised variables were assessed with independent *t*-tests. eGDR, estimated Glucose Disposal Rate; TF activity, Tissue Factor activity; PAI-1, Plasminogen Activator Inhibitor-1; TNFα, Tumour Necrosis Factor alpha. | | | |
